# Supplementary material for: Reusability and composability in process description maps: RAS–RAF–MEK–ERK signalling
Source: Brief Bioinform. 2021 Apr 8;22(5):bbab103. doi: 10.1093/bib/bbab103 (PMC8425390; doi:10.1093/bib/bbab103)

Supplementary Figure S1. The BRAF fragment of the MAPK module from the Cell Survival map of the Atlas of Cancer Signalling Networks (https://acsn.curie.fr) [Kuperstein 2015 PMID:26192618].

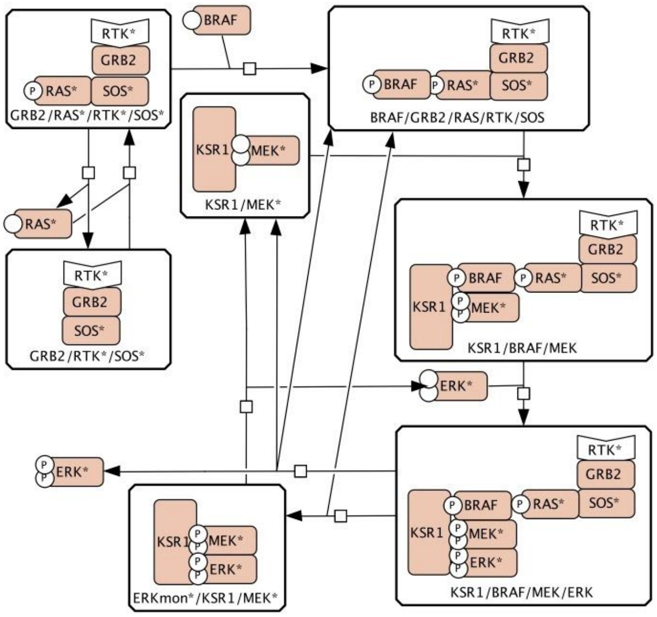

Supplement: Supplementary_Figure_S1_bbab103 [file supplementary_figure_s1_bbab103.pdf]
