# Supplementary material for: Reusability and composability in process description maps: RAS–RAF–MEK–ERK signalling
Source: Brief Bioinform. 2021 Apr 8;22(5):bbab103. doi: 10.1093/bib/bbab103 (PMC8425390; doi:10.1093/bib/bbab103)

Supplementary Figure S2. An inferred CellDesinger's view alternative to Figure 3a - the RAF/MAP Kinase Cascade map (Pathway:R-HSA-5673001) from the Reactome pathway database (https://reactome.org) [Jassal 2020 PMID:31691815; Fabregat 2018 PMID:29377902].

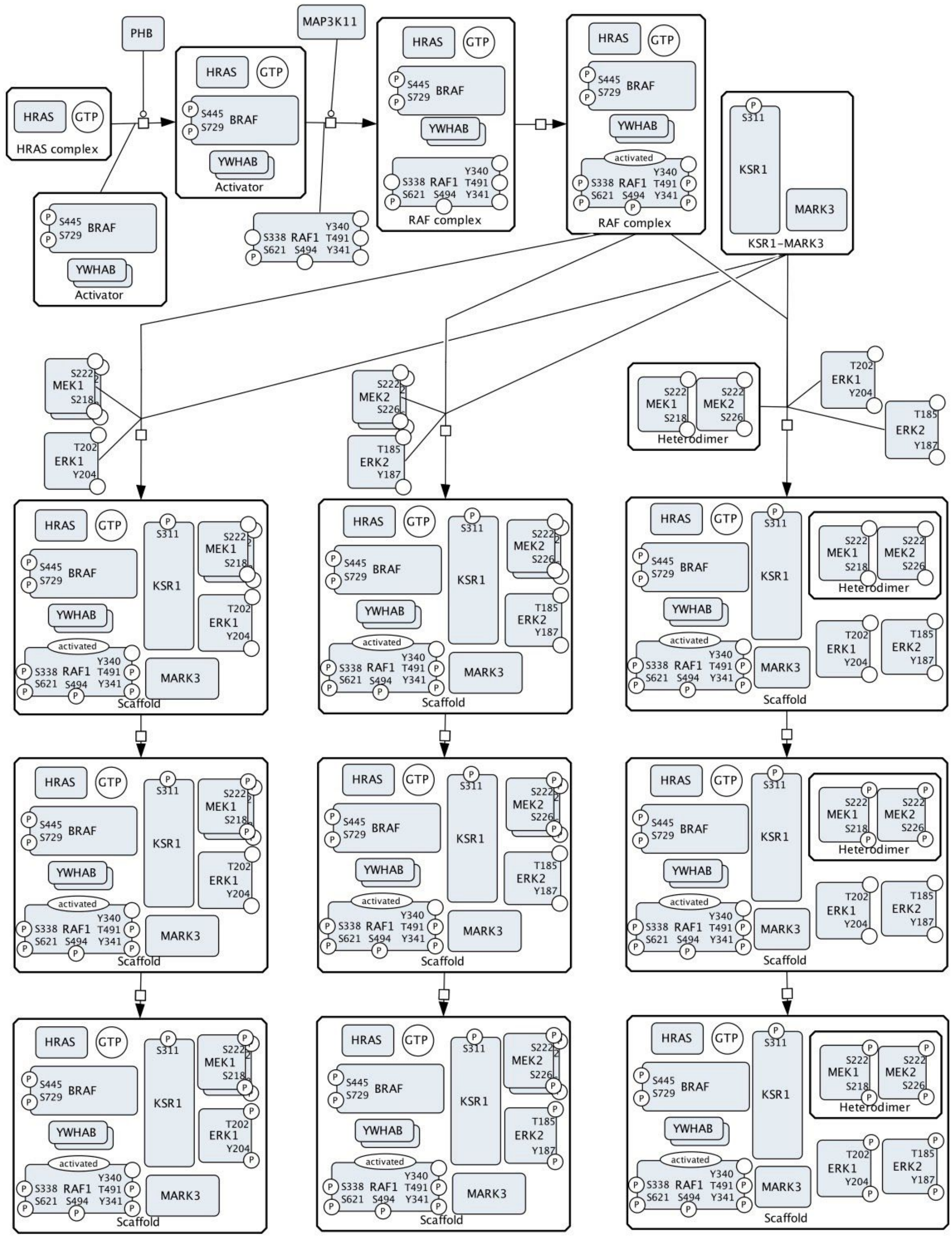

Supplement: Supplementary_Figure_S2_bbab103 [file supplementary_figure_s2_bbab103.pdf]
