# Supplementary material for: Reusability and composability in process description maps: RAS–RAF–MEK–ERK signalling
Source: Brief Bioinform. 2021 Apr 8;22(5):bbab103. doi: 10.1093/bib/bbab103 (PMC8425390; doi:10.1093/bib/bbab103)
Supplement: Supplementary_Table_S1_bbab103 [file supplementary_table_s1_bbab103.pdf]

Supplementary Table S1. Selected features of the maps from the Atlas of Cancer Signalling Network, PANTHER and Reactome databases.

Features	ACSN			PANTHER			Reactome		
	1a	1c	1d	2a	2b	2c	3a	3b	3c
Identifiers provided for all proteins (UniProt)	✓	✓	✓	✓	✓	✓	✓	✓	✓
Identifiers provided for all metabolites (ChEBI)	✓	✓	✓	✓	✓	✓	✓	✓	✓
Each protein modification is described properly in SBGN-compatible format	✓	✓	✓	✓	✓	✓	✓	✓	✓
Each complex composition is reflected in its content in SBGN-compatible format	✓	✓	✓	✓	✓	✓	✓	✓	✓
There are no “mixed” diagrams (no elements of the Reduced Notation of CellDesigner are used)	✓*	✓*	✓*	✓	✓	✓	✓	✓	✓
Available formats: CellDesigner	✓	✓	✓	✓	✓	✓	✗	✗	✗
Available formats: SBGN-ML 0.2	✓	✓	✓	✗	✗	✗	✓	✓	✓
Available formats: SBGN-ML 0.3**	✗	✗	✗	✗	✗	✗	✗	✗	✗
Available formats: SBML***	✓	✓	✓	✓	✓	✓	✓	✓	✓
Available formats: BioPAX Level 2	✗	✗	✗	✗	✗	✗	✓	✓	✓
Available formats: BioPAX Level 3	✗	✗	✗	✓	✓	✓	✓	✓	✓

*Rare cases
**Recently published version with the support for annotation and colours
***With CellDesigner’s XML available it is possible to export the content in SBML Level 1 Version 2 - SBML Level 2 Version 4
